# Serum Vitamin D Levels and Disease Activity in Systemic Lupus Erythematosus: Association with Anti-dsDNA Antibodies and Selected Lifestyle Factors

**DOI:** 10.3390/jcm15135185

**Published:** 2026-07-02

**Authors:** Aleksandra Fijałkowska, Elżbieta Anna Dziankowska-Zaborszczyk, Anna Jolanta Woźniacka

**Affiliations:** 1 Department of Dermatology and Venereology, Medical University of Lodz, 90-647 Lodz, Poland; anna.wozniacka@umed.lodz.pl; 2 Department of Public Health, Epidemiology and Biostatistics, Medical University of Lodz, 90-752 Lodz, Poland; elzbieta.dziankowska-zaborszczyk@umed.lodz.pl

**Keywords:** connective tissue disorders, lupus erythematosus, anti-dsDNA, autoimmunity, vitamin D, therapeutic targets, lifestyle factors

## Abstract

**Background**: Vitamin D is involved not only in calcium–phosphate homeostasis but also in immune and endothelial regulation. Vitamin D deficiency has been suggested to worsen disease activity in systemic lupus erythematosus (SLE). Environmental and lifestyle factors, including seasonal sun exposure, smoking, diet, and supplementation, may influence vitamin D status and disease manifestations. This study aimed to evaluate the association between serum 25-hydroxyvitamin D [25(OH)D] levels, disease activity, and anti-double-stranded DNA (anti-dsDNA) antibody titers in patients with SLE, taking selected lifestyle and environmental factors into account. **Methods**: Serum 25(OH)D concentrations, SLE disease activity assessed by the Systemic Lupus Erythematosus Disease Activity Index 2000 (SLEDAI-2K) score, and anti-dsDNA antibody titers were measured in patients with SLE and healthy controls. Blood samples were collected during sunny (April–September) and non-sunny (October–March) months. Information on vitamin D supplementation, smoking status, and dietary habits was obtained using a structured questionnaire. Associations between vitamin D status, disease activity, anti-dsDNA seropositivity, season of blood collection, supplementation, smoking, and diet were analyzed statistically. **Results**: Patients with SLE had significantly higher mean serum 25(OH)D levels than controls, mainly due to frequent vitamin D supplementation. No significant associations were observed between serum 25(OH)D levels and SLEDAI-2K scores or anti-dsDNA antibody positivity. Seasonality, smoking status, and adherence to special diets were not significantly related to disease activity or anti-dsDNA seropositivity. Vitamin D supplementation was strongly associated with sufficient 25(OH)D levels but did not translate into reduced disease activity or lower anti-dsDNA prevalence. **Conclusions**: Serum 25(OH)D concentration was not associated with clinical or immunological activity of SLE in this cross-sectional study, despite effective correction of deficiency through supplementation. These findings likely reflect the heterogeneity of SLE and the limitations of single time-point assessments, although regular monitoring and individualized vitamin D supplementation may still be considered in SLE care, particularly in the context of recommended photoprotection.

## 1. Introduction

Systemic lupus erythematosus (SLE) is a connective tissue disease with a heterogeneous clinical presentation characterized by periods of exacerbation and remission. Despite the development of advanced laboratory techniques and knowledge of the pathological basis, the exact pathogenesis of the disease is not fully understood. In SLE, exposure to certain environmental factors like ultraviolet B (UVB) radiation, infections, and toxins triggers a loss of immune tolerance in genetically predisposed individuals and leads to abnormal activation of autoimmunity. Self-antigens are exposed to the immune cells which activate innate and adaptive immunity. T lymphocytes secrete pro-inflammatory cytokines that induce B cells to produce antinuclear antibodies (ANA) that play a key role in autoimmune processes in SLE [1,2].

Antibodies directed against double-stranded DNA (anti-dsDNA) deserve special attention, as they are the only ANA whose titers correlate with SLE activity in patients. Anti-dsDNA antibodies are therefore reliable serologic biomarkers included in the SLE disease activity index (SLEDAI 2K) and enable monitoring of treatment effectiveness and rapid detection of disease exacerbation [3,4].

Considering the complex immunopathogenesis of SLE, growing research efforts have focused on factors that modulate the immune response. Over recent decades, vitamin D has been recognized not only for its classical hormonal role in calcium and bone metabolism but also for its significant immunoregulatory properties [5]. Numerous studies have demonstrated that vitamin D exerts immunomodulatory effects by influencing the activity and differentiation of T and B lymphocytes, thereby attenuating autoimmune responses [6]. Conversely, vitamin D deficiency may contribute to excessive B-cell activation and the production of autoantibodies, including anti-dsDNA antibodies. Evidence from the literature indicates a negative correlation between serum vitamin D levels and anti-dsDNA antibody titers [7,8]. Furthermore, several studies suggest that vitamin D supplementation may reduce disease activity and, consequently, decrease autoantibody levels in patients with SLE [7,9].

This study aimed to explore potential associations between serum vitamin D levels, disease activity, and anti-dsDNA antibody status in a real-world cohort of patients with SLE, taking into account selected lifestyle and environmental factors.

## 2. Materials and Methods

A cross-sectional study was conducted at the Department of Dermatology and Venereology at the Medical University of Lodz, Poland. Written informed consent was obtained from all participants prior to the initiation of the study procedures. The study was conducted in accordance with the Declaration of Helsinki and institutional ethical standards and was approved by the Bioethics Committee of the Medical University of Lodz (Approval No. RNN/121/24/KE). The study population consisted of patients diagnosed with SLE according to the 2019 EULAR/ACR classification criteria and a group of healthy volunteers serving as controls.

The study group included 51 patients with SLE: 45 women and 6 men aged from 25 to 78 years who were under the care of the Department of Dermatology and Venereology. The clinical phenotype of SLE patients was heterogeneous and included individuals with predominantly mucocutaneous, musculoskeletal, or systemic manifestations. Because this was a real-world cross-sectional cohort recruited in routine clinical practice, patients were receiving various standard therapies, including antimalarials, glucocorticoids, and immunosuppressive agents according to clinical indications. The healthy control group (*n* = 52, 37 women and 15 men, mean age 51.0) was randomly recruited from patients attending the Dermatology Department with non-autoimmune and non-inflammatory dermatological conditions, hospital staff, and their relatives. The inclusion criteria were age greater than or equal to 18 years and no history of autoimmune diseases. All participants were informed of the study’s purpose and procedures and had the right to withdraw at any time without providing a reason. The control group was included to provide a reference population for comparison of serum vitamin D concentrations and anti-dsDNA antibody prevalence in individuals without autoimmune disease.

Disease activity in patients with SLE was assessed using the SLEDAI-2K. Patients with a SLEDAI-2K score greater than 6 were classified as having high disease activity.

Peripheral venous blood samples (approximately 9 mL) were collected from each participant after an overnight fast of at least 8 h. Vitamin D concentration was determined as serum 25-hydroxyvitamin D using a commercial enzyme-linked immunosorbent assay (ELISA) kit (DRG Instruments GmbH, Marburg, Germany), according to the manufacturer’s instructions.

Vitamin D status was categorized as follows: deficiency: <20 ng/mL, insufficiency: 20–30 ng/mL, sufficiency: >30 ng/mL. The presence of anti-dsDNA antibodies was determined using a semi-quantitative immunoblot assay (EUROLINE ANA Profile (IgG), EUROIMMUN AG, Lübeck, Germany), performed according to the manufacturer’s instructions.

Blood samples were collected once from each participant during routine clinical visits. The timing of blood collection varied throughout the year, and therefore the month of sampling was recorded to account for seasonal variability in vitamin D synthesis. For analytical purposes, months were categorized as “sunny” (April–September) and “non-sunny” (October–March), reflecting seasonal differences in ultraviolet B (UVB) exposure in Poland, which may influence endogenous vitamin D production. The classification was based on long-term climatological data from the Polish Institute of Meteorology and Water Management (IMGW-PIB) and published analyses of sunshine duration in Poland demonstrating substantially higher solar exposure during the April–September period [10,11,12].

In addition, participants in the study had to complete a questionnaire collecting demographic and lifestyle information, including vitamin D supplementation, smoking, and diet. Participants were specifically asked whether they had used vitamin D supplements during the previous year up to the time of blood collection and to report their usual daily dose. Smoking and diet were also taken into account over the past year.

This study had an observational cross-sectional design. Vitamin D supplementation was not introduced as an intervention within the study protocol but reflected routine clinical practice and patient self-reported supplementation. Therefore, participants were categorized according to their current supplementation status for analytical purposes only.

### Statistical Analysis

Data collection and preliminary processing were performed using IBM SPSS Statistics version 27.0 (IBM Corp., Armonk, NY, USA). The Shapiro–Wilk test was used to assess the normality of the distribution of vitamin D levels (measurable variable). Due to the fact that the distribution of vitamin D levels in the SLE group and in the control group deviated from the normal distribution, comparisons for this characteristic between the SLE group and the control group were performed using the two-tailed Mann–Whitney test. Associations between categorical variables, including the presence of anti-dsDNA antibodies and vitamin D status, were examined using the Chi-square test of independence (χ^2^) or Fisher’s exact test, where appropriate.

Correlation analyses between serum vitamin D levels and disease activity scores (SLEDAI-2K) were performed using Spearman’s rank correlation coefficient (ρ). In order to assess the relationship between vitamin D concentration, disease activity and the presence of anti-dsDNA antibodies, univariate logistic regression was also used to determine odds ratios (OR) with 95% confidence intervals (95% CI).

Additionally, the statistical analysis included the assessment of environmental and lifestyle factors, such as the season of blood sample collection (sunny months: April–September vs. non-sunny months: October–March), vitamin D supplementation, cigarette smoking, and adherence to special diets (including lactose-free, gluten-free, low-fat, diabetic, and Mediterranean diets). Associations between these variables and disease activity (SLEDAI-2K score) as well as the presence of anti-dsDNA antibodies were evaluated using the Chi-square (χ^2^) test or Fisher’s exact test, as appropriate, depending on cell counts in contingency tables.

To assess the impact of seasonality of blood collection, vitamin D supplementation, smoking status, and diet on the risk of high disease activity (SLEDAI-2K > 6) and anti-dsDNA seropositivity, univariate logistic regression analyses were performed, with OR and 95% CI calculated. These analyses allowed for the evaluation of the potential influence of the investigated factors as confounding variables in the relationship between serum 25(OH)D concentration and clinical as well as immunological activity of SLE.

The results of analyses for which *p* < 0.05 were considered statistically significant.

## 3. Results

### 3.1. Study Population

To improve the clarity of data presentation, only the key tables essential to the interpretation of the primary findings are included in the main manuscript. Detailed cross-tabulations and secondary analyses are provided in the Supplementary Materials (Supplementary Tables S1–S5).

The study included 51 patients with SLE and 52 healthy controls. Baseline demographic, clinical, treatment-related, and lifestyle characteristics of the study population are presented in Table 1.

The mean serum vitamin D concentration in the SLE group was 29.83 ± 11.92 ng/mL, ranging from 8.67 to 71.50 ng/mL. Among SLE patients supplementing vitamin D (285–5000 IU per day), the average vitamin D level was 33.46 ng/mL, and among those not supplementing, it was 21.88 ng/mL. In the control group, the mean concentration was 23.10 ± 15.66 ng/mL (range: 4.62–86.40 ng/mL). Among individuals in the control group supplementing vitamin D (2000–4000 IU per day), the average vitamin D level was 27.84 ng/mL, while among those not supplementing, it was 17.42 ng/mL.

According to the Mann–Whitney U test, serum vitamin D levels were significantly higher in SLE patients than in controls (Z = 3.72; *p* < 0.001) (Table 2).

### 3.2. Prevalence of Anti-dsDNA Antibodies

Anti-dsDNA antibodies were detected in 15.7% (8/51) of SLE patients, indicating a small number of seropositive individuals in this cohort, and 3.8% (2/52) of controls. This difference was statistically significant (*χ*^2^ = 4.118; *p* = 0.042). Thus, the occurrence of anti-dsDNA positivity was significantly more frequent among patients with SLE compared with healthy individuals (Table 3).

### 3.3. Association Between Disease Activity and the Presence of Anti-dsDNA Antibodies

The analysis showed a statistically significant correlation between disease activity assessed using the SLEDAI-2K scale and the presence of anti-dsDNA antibodies in the group of patients with SLE. Among patients with positive anti-dsDNA antibodies, all patients (100%) were in the low disease activity group (SLEDAI ≤ 6), while no cases of high disease activity (SLEDAI > 6) were reported in this group. In comparison, among patients without anti-dsDNA antibodies, high disease activity was observed in 34.9% of patients. This difference was statistically significant (*p* < 0.05), reflecting an unexpected inverse relationship between anti-dsDNA seropositivity and clinical disease activity in this study population (Table 4).

### 3.4. Association Between Serum Vitamin D Level and Disease Activity

To assess the relationship between vitamin D status and SLE disease activity, patients were stratified by serum vitamin D concentration: below normal (<30 ng/mL) and normal (≥30 ng/mL).

Among patients with normal vitamin D levels, 72.0% showed low and 28.0% high disease activity. The difference was not statistically significant (*χ*^2^ = 0.047; *p* = 0.828) (Table 5).

Spearman’s rank correlation confirmed the absence of a significant linear relationship between serum vitamin D concentration and SLEDAI score (ρ = −0.114; *p* = 0.428). In a univariate logistic regression model, a 1 ng/mL increase in serum vitamin D was associated with a 3% higher odds of low disease activity (OR = 1.025; 95% CI = 0.967–1.085; *p* = 0.381), though this association did not reach statistical significance.

### 3.5. Correlation Between Vitamin D Levels and Anti-dsDNA Antibody Positivity

In the SLE group, anti-dsDNA antibodies were detected in 15.7% of patients. Among those with low vitamin D levels (<30 ng/mL), only 7.7% were dsDNA-positive, compared to 24.0% in the group with normal vitamin D levels. The difference was not statistically significant (*χ*^2^ = 2.563; *p* = 0.109) (Table 6).

Univariate logistic regression showed that each 1 ng/mL increase in vitamin D was associated with a 4% higher odds of anti-dsDNA negativity (OR = 1.042; 95% CI = 0.979–1.110; *p* = 0.191), but the result was not statistically significant.

## 4. Discussion

Vitamin D has attracted considerable interest in SLE research because of its immunomodulatory and endothelial-protective properties [13]. The epidermis contains a natural precursor of cholecalciferol, 7-dehydrocholesterol, which in the liver undergoes further conversion to inactive 25-hydroxyvitamin D (25(OH)D), then converted to biologically active 1,25-dihydroxyvitamin D (1,25(OH)_2_D) [14,15]. Through activation of the vitamin D receptor (VDR), it influences both innate and adaptive immune responses and may contribute to the maintenance of immune tolerance. Experimental and clinical studies have therefore suggested that vitamin D deficiency may be associated with increased disease activity and enhanced autoantibody production in SLE [14,16]. Based on these observations, we hypothesized that lower serum 25(OH)D concentrations would be associated with higher disease activity, as assessed by the SLEDAI-2K score, and with a higher prevalence of anti-dsDNA seropositivity.

In our study cohort, patients with SLE presented with a significantly higher mean serum 25(OH)D concentration compared with healthy controls, despite the high prevalence of vitamin D insufficiency expected in autoimmune diseases. This difference is most likely explained by the relatively higher prevalence of vitamin D supplementation among SLE patients compared with controls (35 vs. 23). Routine prophylactic or therapeutic supplementation is increasingly recommended in dermatologic and rheumatologic practice to counteract iatrogenic deficiencies associated with widely recommended photoprotection, exposure to glucocorticoids, renal insufficiency and limited outdoor activity due to inflammatory joint and muscle pain among SLE patients [17]. Importantly, in this cohort, serum vitamin D levels were primarily determined by supplementation rather than by disease-related biological mechanisms, which substantially limits their interpretability as disease-related biomarkers. Therefore, comparisons between SLE patients and controls in terms of vitamin D concentration should not be interpreted as reflecting intrinsic disease-specific differences.

As predicted, anti-dsDNA antibodies were found at a considerably higher frequency among SLE patients than among healthy individuals, confirming the serological distinction between the groups. The inverse association observed in our cohort between anti-dsDNA seropositivity and lower disease activity should be interpreted with caution. First, the very small number of anti-dsDNA-positive patients (*n* = 8) severely limits the reliability of any observed associations and makes the corresponding estimates highly unstable. Second, most patients were receiving ongoing immunomodulatory treatment, which may suppress clinical manifestations of the disease while autoantibody positivity persists. Third, anti-dsDNA antibodies in this study were assessed using a semi-quantitative immunoblot assay (EUROLINE ANA profile), which allows detection of antibody reactivity but does not provide precise quantitative titers. Compared with quantitative assays such as ELISA or the Farr assay, this method may have lower sensitivity for detecting variations in antibody levels that correlate with disease activity. Consequently, the unexpected inverse association observed in this cross-sectional analysis is most likely explained by methodological and sample-size limitations rather than by a true biological relationship. However, serum 25(OH)D levels did not correlate with disease activity expressed as the SLEDAI-2K score. Also, no significant association was observed between vitamin D concentrations and anti-dsDNA antibody titers. The results suggest that vitamin D levels—at least under the real-world conditions of routine clinical supplementation—do not represent reliable indicators of immunological or clinical activity in SLE [17].

Given the central role of UVB radiation in cutaneous vitamin D synthesis, we also assessed whether the season of blood sample collection influenced disease activity or anti-dsDNA antibody presence. Although high disease activity was more frequently observed during sunny months compared with non-sunny months, this association did not reach statistical significance (Supplementary Table S1). Similarly, no significant seasonal differences were found in anti-dsDNA seropositivity (Supplementary Table S2).

These findings may appear counterintuitive, as increased sun exposure is classically associated with higher vitamin D levels [18]. However, in patients with SLE, sunlight exposure represents a complex factor, as ultraviolet radiation is a well-established trigger of disease flares [19,20]. Consequently, increased disease activity during sunny months may reflect UV-induced immunological activation rather than beneficial effects mediated by vitamin D synthesis [18,21]. This dual and opposing role of sunlight likely attenuates any straightforward seasonal relationship between vitamin D status and disease activity in SLE [19,22].

Our additional analyses demonstrated that vitamin D supplementation was strongly associated with achieving normal serum 25(OH)D concentrations, increasing the odds more than sevenfold. However, despite its clear biochemical effectiveness, supplementation was not associated with lower disease activity or reduced anti-dsDNA seropositivity.

Vitamin D supplementation is frequently initiated in patients with more severe disease or long disease duration, which may introduce confounding by indication. Moreover, the immunomodulatory effects of vitamin D are likely to be subtle and cumulative. Therefore, supplementation may contribute more to long-term disease modulation and prevention of flares than to short-term reductions in SLEDAI-2K scores or autoantibody titers.

Additionally, CRP was analyzed as a marker of systemic inflammation. No significant correlations were observed between CRP and serum 25(OH)D concentration (ρ = −0.063, *p* = 0.662; Supplementary Table S12) or between CRP and SLEDAI-2K score (ρ = 0.009, *p* = 0.952; Supplementary Table S12). Similarly, anti-dsDNA seropositivity was not significantly associated with elevated CRP levels (>5 mg/L) (*p* = 0.785; Supplementary Table S13). These findings further support the absence of a clear relationship between vitamin D status, inflammatory markers and disease activity in this cohort.

Cigarette smoking has been previously identified as a potential modifier of autoimmune disease activity through its pro-inflammatory, oxidative, and epigenetic effects [23,24,25]. In our cohort, smokers tended to exhibit higher disease activity compared with non-smokers (40.0% vs. 25.0%), although this difference did not reach statistical significance (Supplementary Table S8). Similarly, smoking was not associated with anti-dsDNA antibody positivity (Supplementary Table S9). Importantly, cigarette smoking was also reported among SLE patients receiving antimalarial therapy, and tobacco exposure may represent an additional factor potentially associated with reduced treatment response in this subgroup [26].

The lack of statistically significant associations in our study may be explained by the relatively small sample size and the heterogeneous smoking history among participants. Nevertheless, the observed trend toward higher disease activity in smokers is consistent with prior reports suggesting that smoking may impair immune regulation in SLE and ultimately cause increased disease activity. For example, Ghaussy et al. demonstrated higher disease activity among smokers with SLE [27], whereas Speyer and Costenbader highlighted smoking as a potential environmental factor contributing to lupus pathogenesis and disease severity [25]. Although our results did not reach statistical significance, the direction of the observed association was consistent with these reports. These findings support the continued recommendation of smoking cessation as part of comprehensive disease management [27,28,29].

Dietary factors have been increasingly investigated as potential modulators of immune function and systemic inflammation in autoimmune diseases [30,31]. In our study, adherence to special diets, including lactose-free, gluten-free, low-fat, diabetic, and Mediterranean diets, was not associated with differences in disease activity or anti-dsDNA antibody prevalence.

It should be noted, however, that dietary effects are often difficult to quantify due to variability in adherence, duration, and nutritional composition. Moreover, dietary interventions may primarily affect metabolic health, fatigue, or quality of life rather than directly modifying immunological disease activity [32,33].

Importantly, the cross-sectional design of this study may not fully capture the dynamic relationship between vitamin D status and immunological activity in SLE. Additional evidence for the complexity of vitamin D metabolism in SLE was provided by Meza-Meza et al., who demonstrated that patients with active or renal disease may exhibit low serum 25(OH)D levels despite elevated calcitriol concentrations and an increased calcitriol-to-calcidiol ratio. These findings suggest altered vitamin D metabolism during inflammatory states and highlight the limitations of relying solely on 25(OH)D as a marker of vitamin D status in SLE [34].

Several previous studies have reported an inverse relationship between serum 25(OH)D levels and SLE disease activity, with lower vitamin D concentrations associated with higher SLEDAI-2K scores and increased anti-dsDNA antibody levels. In addition, some prospective and interventional studies have suggested that vitamin D supplementation may reduce disease activity and anti-dsDNA levels, supporting a potential role of vitamin D in the modulation of autoimmune responses [35,36].

Recent meta-analyses provide important context for interpreting our findings. A systematic review and meta-analysis by El Kababi et al. reported that vitamin D supplementation was associated with modest reductions in SLE disease activity (SLEDAI-2K) in a proportion of randomized and interventional studies, although results varied depending on supplementation dose, disease duration, and study design [35]. Similarly, the meta-analysis by Irfan et al. demonstrated improvement in overall disease activity, while changes in immunological markers such as anti-dsDNA antibodies and complement levels remained inconsistent [36]. These observations are consistent with our results, in which serum vitamin D levels were not significantly associated with anti-dsDNA seropositivity.

Studies conducted in different ethnic populations have also reported inconsistent findings. Investigations performed in Chinese, Indian, Australian, Egyptian, and Saudi cohorts generally demonstrated an inverse association between serum 25(OH)D concentrations and disease activity, with lower vitamin D levels observed in patients with more active disease [8,37,38,39,40]. Similarly, Arshad et al. reported a significant association between vitamin D deficiency and increased disease activity in a Pakistani cohort, both at diagnosis and during follow-up [41]. In contrast, García-Carrasco et al. found no association between vitamin D levels and disease activity in Mexican patients with SLE [42], while Kavadichanda et al. observed only a weak inverse correlation that was no longer clinically relevant after adjustment for confounding factors [38]. These discrepancies may reflect differences in ethnicity, geographic location, sunlight exposure, supplementation practices, disease phenotype, treatment regimens, and study design. Our findings are therefore consistent with the growing body of evidence indicating that the relationship between vitamin D status and SLE activity is complex and may vary across populations.

From a clinical point of view, regular monitoring of serum 25(OH)D concentrations and individual adjustment of supplementation, especially in younger patients with photosensitivity, those treated with glucocorticoids, or those who limit their outdoor exposure, may still be recommended. Therapeutically, daily doses in the range of 2000–4000 IU are generally considered safe for long-term supplementation, whereas higher doses (5000–7000 IU/day or 50,000 IU/week for 8–12 weeks) may be used temporarily under specialist supervision to correct significant deficiency. Supplementation should be individualized according to baseline levels, body mass index, and comorbidities. Such an approach may help reduce fatigue, support endothelial function, and potentially attenuate immune overactivation in SLE [35,36]. Furthermore, in population-based studies, serum 25(OH)D concentrations in the range of approximately 40–60 ng/mL have been associated with the lowest risk of autoimmune diseases and all-cause mortality [43,44].

This study has several limitations that should be considered when interpreting the results. Firstly, a key limitation is the low statistical power of several analyses. Post hoc power calculations for the Chi-square tests indicated very low power (typically 2–30%) for most associations, except for the strong effect of vitamin D supplementation on serum 25(OH)D levels. The low number of outcome events (only 15 cases with high SLEDAI-2K and 8 patients positive for anti-dsDNA antibodies) resulted in insufficient events-per-variable (EPV) for logistic regression, making even univariate models statistically fragile and their estimates unstable. Therefore, the non-significant results should be interpreted with caution, as the study may not have been able to detect existing associations. A multivariate logistic regression was not feasible due to the very small number of outcome events, resulting in an events-per-variable ratio far below the recommended threshold. This would make any multivariable model statistically unstable. Potential confounders (age, sex, smoking, diet, season, vitamin D supplementation) were examined individually, but none showed significant associations with the outcomes, and post hoc power was low (2–30%). Therefore, multivariable adjustment would not have produced reliable estimates, and all regression findings should be interpreted as exploratory. Furthermore, its cross-sectional character allows only for assessment at a single point in time, which limits the possibility of establishing a cause-and-effect relationship between vitamin D levels and disease activity. Another limitation of this study is the clinical heterogeneity of the SLE cohort. Patients presented different disease phenotypes and were receiving various therapies in routine clinical practice (Table 1), which may have obscured potential associations. An additional descriptive analysis examining the relationship between 25(OH)D levels, disease phenotype, and medication exposure confirmed substantial heterogeneity within the study population. Vitamin D concentrations across different organ domains (cutaneous, articular, renal, hematologic, or immunological) ranged widely between 18 and 42 ng/mL, showing no clear trend (Supplementary Table S10). Similarly, mean vitamin D levels among patients receiving various treatment regimens (antimalarial drugs, glucocorticoids, immunosuppressants) were comparable, and the small sample size in each subgroup precluded reliable comparative analyses (Supplementary Table S11). Previous studies have suggested that vitamin D deficiency may be particularly common in patients with cutaneous lupus erythematosus, especially among those practicing strict photoprotection [45].

The sex distribution also differed between the SLE and control groups, reflecting the well-known female predominance of SLE. This difference should be considered when interpreting comparisons between groups. Hormonal status and sex hormone concentrations were not assessed in our study. Although vitamin D metabolism may be influenced by endocrine factors, the small number of male patients precluded meaningful sex-stratified analyses.

Another limitation concerns the composition of the control group. Healthy controls were recruited from dermatology outpatients with non-autoimmune conditions, hospital staff, and their relatives, which may introduce a degree of heterogeneity and potential selection bias. In addition, a small proportion of control participants (3.8%) showed anti-dsDNA positivity. Although all controls had no clinical history of autoimmune disease, this finding may reflect the limited specificity of semi-quantitative immunoblot assays or the possibility of low-level autoantibody reactivity in otherwise healthy individuals. The absence of systematic ANA screening prior to inclusion may also have contributed to this observation.

A key aspect that should also be considered when interpreting our findings is the distinction between short-term disease activity and long-term disease outcomes. In this study, disease activity was assessed using the SLEDAI-2K score, which captures current inflammatory manifestations but does not reflect cumulative organ damage, subclinical disease activity, fatigue, or quality of life. It is therefore conceivable that vitamin D may exert more pronounced effects on long-term disease trajectories, endothelial function, or damage accrual rather than on point-in-time activity indices.

Another limitation of the present study is the lack of molecular profiling analyses. Recent multi-omics approaches integrating transcriptomics, single-cell sequencing, and immune-cell phenotyping have demonstrated their ability to uncover complex immunoregulatory mechanisms and identify clinically relevant biomarkers in immune-mediated diseases. Future studies combining clinical assessment with transcriptomic, proteomic, metabolomic, or single-cell approaches may provide a more comprehensive understanding of the immunomodulatory effects of vitamin D in SLE and help explain the heterogeneity of disease activity and treatment response [46].

Recent evidence suggests that vitamin D may exert protective effects against oxidative stress and DNA damage through multiple molecular pathways. A recent systematic review demonstrated that vitamin D deficiency is frequently associated with increased markers of DNA damage and oxidative stress, whereas supplementation may attenuate these processes. Although such mechanisms were not evaluated in the present study, they may partly explain the complex relationship between vitamin D status and immune dysregulation in SLE and warrant further investigation [47].

From a clinical perspective, our findings support the view that serum 25(OH)D concentration should not be regarded as a reliable standalone biomarker of disease activity in patients with established SLE receiving routine care. Nevertheless, given the well-documented immunomodulatory, endothelial-protective, and musculoskeletal benefits of vitamin D, regular monitoring and individualized supplementation may still be considered as part of general supportive care in patients with SLE.

## 5. Conclusions

In conclusion, serum 25(OH)D concentrations were not significantly associated with disease activity assessed by SLEDAI-2K or with anti-dsDNA seropositivity in this cohort of patients with SLE. Vitamin D supplementation was effective in improving vitamin D status but was not associated with reduced clinical or immunological disease activity. Given the cross-sectional design, widespread supplementation, and limited statistical power, these findings should be considered exploratory and hypothesis-generating. Further prospective studies are needed to clarify the role of vitamin D in SLE pathogenesis and disease modulation.

## Figures and Tables

**Table 1:** Baseline demographic, clinical, treatment-related, and lifestyle characteristics of the research group and healthy controls. Abbreviations: SLE, systemic lupus erythematosus; SD, standard deviation; UVB, ultraviolet B radiation. ^†^ Other immunosuppressive therapy includes azathioprine (AZA), methotrexate (MTX), and mycophenolate mofetil (MM); ^‡^ Special diet included lactose-free, gluten-free, low-fat, diabetic, and Mediterranean diets; - Not applicable.

Characteristic	SLE (*n* = 51)	Controls (*n* = 52)
Age, years (mean ± SD)	49.8 ± 12.6	51.0 ± 15.4
Female sex, *n* (%)	44 (86.3)	37 (71.2)
Male sex, *n* (%)	7 (13.7)	15 (28.8)
Vitamin D supplementation, *n* (%)	35 (68.6)	23 (44.2)
Vitamin D dose, IU/day, range	285–5000	2000–4000
Smokers, *n* (%)	15 (29.84)	9 (17.3)
Special diet ^‡^, *n* (%)	7 (13.7)	5 (9.6)
Blood collection during high-UVB months, *n* (%)	30 (58.8)	28 (53.8)
Blood collection during low-UVB months, *n* (%)	21 (41.2)	24 (46.2)
Immunological involvement, *n* (%)	26 (51.0)	-
Cutaneous involvement, *n* (%)	24 (47.1)	-
Articular involvement, *n* (%)	12 (23.5)	-
Hematological involvement, *n* (%)	11 (21.6)	-
Renal involvement, *n* (%)	3 (5.9)	-
Muscular involvement, *n* (%)	1 (2.0)	-
Ocular involvement, *n* (%)	1 (2.0)	-
Antimalarial therapy, *n* (%)	38 (73.1)	-
Glucocorticoid therapy, *n* (%)	29 (56.9)	-
Other immunosuppressive therapy ^†^, *n* (%)	18 (35.3)	-

**Table 2:** Comparison of serum vitamin D levels between patients with SLE and healthy controls. Abbreviations: SD—standard deviation; Min—minimum; Max—maximum.

Group	N	Mean (ng/mL)	Median	Min	Max	SD	*p*
SLE	51	29.83	29.20	8.67	71.50	11.92	*p* < 0.001
Control	52	23.10	19.70	4.62	86.40	15.66	

**Table 3:** Frequency of anti-dsDNA antibody positivity in patients with SLE and healthy controls.

Group	*n* (Negative)	% (Negative)	*n* (Positive)	% (Positive)	*p*
SLE	43	84.3	8	15.7	0.042
Control	50	96.2	2	3.8	

**Table 4:** Association between anti-dsDNA antibody positivity and SLEDAI-2K.

Activity (SLEDAI-2K Score)	dsDNA					
	Negative		Positive		*p*	
	*n*	%	*n*	%		
Low (≤6)	28	65.1	8	100.0	0.048	
High (>6)	15	34.9	0	0.0		
Total	43	100.0	8	100.0		

**Table 5:** Association between serum vitamin D status and SLEDAI-2K score.

SLEDAI-2K Score	Vitamin D < 30 ng/mL	Vitamin D ≥ 30 ng/mL	$\chi^{2}$	*p*
Low (≤6)	18 (69.2%)	18 (72.0%)	0.047	0.828
High (>6)	8 (30.8%)	7 (28.0%)		

**Table 6:** Association between serum vitamin D status and anti-dsDNA antibody positivity in patients with SLE.

dsDNA Status	Vitamin D < 30 ng/mL	Vitamin D ≥ 30 ng/mL	$\chi^{2}$	*p*
Negative	24 (92.3%)	19 (76.0%)	2.563	0.109
Positive	2 (7.7%)	6 (24.0%)		

## Data Availability

The original contributions presented in this study are included in the article. Further inquiries can be directed to the corresponding author.

## Supplementary Materials

The following supporting information can be downloaded at https://www.mdpi.com/article/10.3390/jcm15135185/s1. Table S1. Association between the month of blood collection (sunny vs. non sunny) and SLEDAI 2K score; Table S2. Association between the month of blood collection (sunny vs. non sunny) and anti dsDNA seropositivity; Table S3. Association between vitamin D supplementation and its concentration in patients with SLE; Table S4. Association between vitamin D supplementation and SLEDAI-2K score; Table S5. Association between vitamin D supplementation and anti-dsDNA antibody positivity in patients with SLE; Table S6. Association between diet and SLEDAI-2K score in patients with SLE; Table S7. Association between diet and anti-dsDNA antibody positivity in patients with SLE; Table S8. Association between smoking and SLEDAI-2K score; Table S9. Association between smoking and anti-dsDNA antibody positivity in patients with SLE; Table S10. Serum vitamin D levels across clinical phenotypes of SLE; Table S11. Serum vitamin D levels according to treatment regimens. Abbreviations: AM—antimalarial drugs, GKS—glucocorticoids, MM—mycophenolate mofetil, AZA—azathioprine, MTX—methotrexate; Table S12. Spearman correlation analysis of serum CRP concentration with serum 25(OH)D levels and SLEDAI-2K score in patients with SLE; Table S13. Association of elevated CRP levels (>5 mg/L) with anti-dsDNA seropositivity in patients with SLE.

## Abbreviations

The following abbreviations are used in this manuscript:

SLE	systemic lupus erythematosus
25(OH)D	25-hydroxyvitamin D
anti-dsDNA	Anti-double-stranded DNA
SLEDAI-2K	Systemic Lupus Erythematosus Disease Activity Index 2000
UVB	ultraviolet B radiation
ANA	antinuclear antibodies
ELISA	enzyme-linked immunosorbent assay
OR	odds ratios
95% CI	95% confidence intervals
IU	international units
SD	standard deviation
Min	minimum
Max	maximum
1,25(OH)2D	1,25-dihydroxyvitamin D
VDR	vitamin D receptor
MHC	major histocompatibility complex
TNF-α	tumor necrosis factor alpha

## References

[1] Ameer M.A., Chaudhry H., Mushtaq J., Khan O.S., Babar M., Hashim T., Zeb S., Tariq M.A., Patlolla S.R., Ali J. (2022). An Overview of Systemic Lupus Erythematosus (SLE) Pathogenesis, Classification, and Management. Cureus.

[2] Crow M.K. (2023). Pathogenesis of Systemic Lupus Erythematosus: Risks, Mechanisms and Therapeutic Targets. Ann. Rheum. Dis..

[3] Hirohata S., Sakuma Y., Yanagida T., Yoshio T. (2014). Association of Cerebrospinal Fluid Anti-Sm Antibodies with Acute Confusional State in Systemic Lupus Erythematosus. Arthritis Res. Ther..

[4] Janwityanuchit S., Verasertniyom O., Vanichapuntu M., Vatanasuk M. (1993). Anti-Sm: Its Predictive Value in Systemic Lupus Erythematosus. Clin. Rheumatol..

[5] Ghaseminejad-Raeini A., Ghaderi A., Sharafi A., Nematollahi-Sani B., Moossavi M., Derakhshani A., Sarab G.A. (2023). Immunomodulatory Actions of Vitamin D in Various Immune-Related Disorders: A Comprehensive Review. Front. Immunol..

[6] Daryabor G., Gholijani N., Kahmini F.R. (2023). A Review of the Critical Role of Vitamin D Axis on the Immune System. Exp. Mol. Pathol..

[7] Franco A.S., Freitas T.Q., Bernardo W.M., Pereira R.M.R. (2017). Vitamin D Supplementation and Disease Activity in Patients with Immune-Mediated Rheumatic Diseases: A Systematic Review and Meta-Analysis. Medicine.

[8] Attar S.M., Siddiqui A.M. (2013). Vitamin d Deficiency in Patients with Systemic Lupus Erythematosus. Oman Med. J..

[9] Magro R., Saliba C., Camilleri L., Scerri C., Borg A.A. (2021). Vitamin D Supplementation in Systemic Lupus Erythematosus: Relationship to Disease Activity, Fatigue and the Interferon Signature Gene Expression. BMC Rheumatol..

[10] Institute of Meteorology and Water Management—National Research Institute (IMGW-PIB) Climate Normals in Poland. https://klimat.imgw.pl/pl/climate-normals/USL.

[11] Institute of Meteorology and Water Management—National Research Institute (IMGW-PIB) Solar Atlas of Poland—Monthly Mean Direct Solar Radiation on a Horizontal Surface. https://klimat.imgw.pl/pl/solar-atlas/#sid/Monthly/1991/1/02/Monthly_mean/.

[12] Bartoszek K., Matuszko D., Węglarczyk S. (2021). Trends in sunshine duration in Poland (1971–2018). Int. J. Climatol..

[13] Akimbekov N.S., Digel I., Sherelkhan D.K., Razzaque M.S. (2022). Vitamin D and Phosphate Interactions in Health and Disease. Adv. Exp. Med. Biol..

[14] Sîrbe C., Rednic S., Grama A., Pop T.L. (2022). An Update on the Effects of Vitamin D on the Immune System and Autoimmune Diseases. Int. J. Mol. Sci..

[15] Charoenngam N., Holick M.F. (2020). Immunologic Effects of Vitamin D on Human Health and Disease. Nutrients.

[16] Sun J., Zhang Y.G. (2022). Vitamin D Receptor Influences Intestinal Barriers in Health and Disease. Cells.

[17] Mok C.C. (2013). Vitamin D and Systemic Lupus Erythematosus: An Update. Expert Rev. Clin. Immunol..

[18] Cardelli C., Zucchi D., Elefante E., Signorini V., Menchini M., Stagnaro C., Mosca M., Tani C. (2024). Environment and Systemic Lupus Erythematosus. Clin. Exp. Rheumatol..

[19] Klein B., Kunz M. (2022). Current Concepts of Photosensitivity in Cutaneous Lupus Erythematosus. Front. Med..

[20] Corbin D., Christian L., Rapp C.M., Liu L., Rohan C.A., Travers J.B. (2023). New Concepts on Abnormal UV Reactions in Systemic Lupus Erythematosus and a Screening Tool for Assessment of Photosensitivity. Ski. Res. Technol..

[21] Dai X., Fan Y., Zhao X. (2025). Systemic Lupus Erythematosus: Updated Insights on the Pathogenesis, Diagnosis, Prevention and Therapeutics. Signal Transduct. Target. Ther..

[22] Tanner T.I., Agalliu I., Wahezi D.M., Rubinstein T.B. (2024). Relationship of Regional Ultraviolet Index Data with Rash and Systemic Disease Activity in Youth with Childhood-Onset Systemic Lupus: Results from the Childhood Arthritis and Rheumatology Research Alliance Registry. Pediatr. Rheumatol. Online J..

[23] Arnson Y., Shoenfeld Y., Amital H. (2010). Effects of Tobacco Smoke on Immunity, Inflammation and Autoimmunity. J. Autoimmun..

[24] Qiu F., Liang C.L., Liu H., Zeng Y.Q., Hou S., Huang S., Lai X., Dai Z. (2017). Impacts of Cigarette Smoking on Immune Responsiveness: Up and down or Upside Down?. Oncotarget.

[25] Speyer C.B., Costenbader K.H. (2018). Cigarette Smoking and the Pathogenesis of Systemic Lupus Erythematosus. Expert Rev. Clin. Immunol..

[26] Jewell M.L., McCauliffe D.E. (2000). Patients with Cutaneous Lupus Erythematosus Who Smoke Are Less Responsive to Antimalarial Treatment. J. Am. Acad. Dermatol..

[27] Ghaussy N.O., Sibbitt W., Bankhurst A.D., Qualls C.R. (2003). Cigarette Smoking and Disease Activity in Systemic Lupus Erythematosus. J. Rheumatol..

[28] Turchin I., Bernatsky S., Clarke A.E., St-Pierre Y., Pineau C.A. (2009). Cigarette Smoking and Cutaneous Damage in Systemic Lupus Erythematosus. J. Rheumatol..

[29] Raymond W.D., Hamdorf M., Furfaro M., Eilertsen G.O., Nossent J.C. (2021). Smoking Associates with Increased BAFF and Decreased Interferon-γ Levels in Patients with Systemic Lupus Erythematosus. Lupus Sci. Med..

[30] Constantin M., Nita I., Olteanu R., Constantin T., Bucur S., Matei C., Raducan A. (2019). Significance and Impact of Dietary Factors on Systemic Lupus Erythematosus Pathogenesis. Exp. Ther. Med..

[31] Aparicio-Soto M., Sánchez-Hidalgo M., Alarcón-De-La-Lastra C. (2017). An Update on Diet and Nutritional Factors in Systemic Lupus Erythematosus Management. Nutr. Res. Rev..

[32] Pardali E.C., Gkouvi A., Gkouskou K.K., Manolakis A.C., Tsigalou C., Goulis D.G., Bogdanos D.P., Grammatikopoulou M.G. (2025). Autoimmune Protocol Diet: A Personalized Elimination Diet for Patients with Autoimmune Diseases. Metabol. Open.

[33] Knippenberg A., Robinson G.A., Wincup C., Ciurtin C., Jury E.C., Kalea A.Z. (2022). Plant-Based Dietary Changes May Improve Symptoms in Patients with Systemic Lupus Erythematosus. Lupus.

[34] Meza-Meza M.R., Muñoz-Valle J.F., Ruiz-Ballesteros A.I., Vizmanos-Lamotte B., Parra-Rojas I., Martínez-López E., Oregon-Romero E., Márquez-Sandoval Y.F., Cerpa-Cruz S., de la Cruz-Mosso U. (2021). Association of High Calcitriol Serum Levels and Its Hydroxylation Efficiency Ratio with Disease Risk in SLE Patients with Vitamin D Deficiency. J. Immunol. Res..

[35] El Kababi S., El Ouali E.M., Kartibou J., Lamiri A., Deblij S., Supriya R., Saiedi A., Del Coso J., Laher I., Zouhal H. (2025). A Systematic Review and Meta-Analysis of the Effects of Vitamin D on Systemic Lupus Erythematosus. Nutrients.

[36] Irfan S.A., Ali A.A., Shabbir N., Altaf H., Ahmed A., Thamara Kunnath J., Divya Boorle N.V.L., Miguel A.K., Loh C.C., Gandrakota N. (2022). Effects of Vitamin D on Systemic Lupus Erythematosus Disease Activity and Autoimmunity: A Systematic Review and Meta-Analysis. Cureus.

[37] Gao C.-C., Liu S.-Y., Wu Z.-Z., Li T.-F., Gao G.-M., Liu Z.-S., Zheng Z.-H. (2016). Severe Vitamin D Deficiency Increases the Risk for Moderate to Severe Disease Activity in Chinese Patients with SLE. Lupus.

[38] Kavadichanda C., Singh P., Maurya S., Tota S., Kiroubagarin A., Kounassegarane D., Anand S., Negi V.S., Aggarwal A. (2023). Clinical and Serological Association of Plasma 25-Hydroxyvitamin D (25(OH)D) Levels in Lupus and the Short-Term Effects of Oral Vitamin D Supplementation. Arthritis Res. Ther..

[39] Yap K.S., Northcott M., Hoi A.B.Y., Morand E.F., Nikpour M. (2015). Association of Low Vitamin D with High Disease Activity in an Australian Systemic Lupus Erythematosus Cohort. Lupus Sci. Med..

[40] Hamza R.T., Awwad K.S., Ali M.K., Hamed A.I. (2011). Reduced Serum Concentrations of 25-Hydroxy Vitamin D in Egyptian Patients with Systemic Lupus Erythematosus: Relation to Disease Activity. Med. Sci. Monit..

[41] Arshad A., Mahmood S.B.Z., Ayaz A., Manji A.A.K., Ahuja A.K. (2021). Association of Vitamin D Deficiency and Disease Activity in Systemic Lupus Erythematosus Patients: Two-Year Follow-Up Study. Arch. Rheumatol..

[42] García-Carrasco M., Mendoza-Pinto C., Etchegaray-Morales I., Soto-Santillán P., Jiménez-Herrera E.A., Robles-Sánchez V., Rodríguez-Gallegos A., Ramos-Varela A., Muñoz-Guarneros M., Ruiz-Argüelles A. (2017). Vitamin D Insufficiency and Deficiency in Mexican Patients with Systemic Lupus Erythematosus: Prevalence and Relationship with Disease Activity. Reumatol. Clin..

[43] Luxwolda M.F., Kuipers R.S., Kema I.P., Van Der Veer E., Dijck-Brouwer D.A.J., Muskiet F.A.J. (2013). Vitamin D status indicators in indigenous populations in East Africa. Eur. J. Nutr..

[44] Wacker M., Holick M.F. (2013). Sunlight and Vitamin D: A global perspective for health. Derm.-Endocrinol..

[45] Cusack C., Danby C., Fallon J.C., Ho W.L., Murray B., Brady J., O’KElly P., Ambrose N., Kearns G., Murphy G.M. (2008). Photoprotective behaviour and sunscreen use: Impact on vitamin D levels in cutaneous lupus erythematosus. Photodermatol. Photoimmunol. Photomed..

[46] Tu B., Fang R., Lu P., Liu M., Yang S., Hu D., Ruan R., Ning R. (2025). Multi-Omic Profiling Reveals Age-Specific Blood Biomarkers and Aging-Driven B Cell Remodeling in Osteoarthritis. Int. J. Surg..

[47] Lestari M.I., Murti K., Iche Andriyani Liberty I.A., Hafy Z., Linardi V., Khoirudin M., Umar T.P. (2023). Association of Vitamin D with Deoxyribonucleic Acid (DNA) Damage: A Systematic Review of Animal and Human Studies. Acta Biochim. Pol..

