# Supplementary material for: Serum Vitamin D Levels and Disease Activity in Systemic Lupus Erythematosus: Association with Anti-dsDNA Antibodies and Selected Lifestyle Factors
Source: J Clin Med. 2026 Jul 2;15(13):5185. doi: 10.3390/jcm15135185 (PMC13363294; doi:10.3390/jcm15135185)
Supplement: Supplementary file 1 [file jcm-15-05185-s001.zip › jcm-4353571-supplementary.pdf]

1. *The effect of the month of blood collection on disease activity and the presence of anti-dsDNA antibodies*

Patients were divided into two groups depending on the season when blood samples were collected: sunny months (April–September) and non-sunny months (October–March). During the sunny period, high disease activity (SLEDAI > 6) occurred more frequently than during the non-sunny period (36.7% vs 19.1%), but this difference was not statistically significant ($\chi^2 = 1.850$; $p > 0.05$). Univariate logistic regression analysis showed that blood sampling during sunny months was associated with a nearly 2.5-fold higher risk of high disease activity (OR = 2.46; 95% CI: 0.637–9.507), but this result was also not statistically significant ($p = 0.187$) (Supplementary Table S1).

Supplementary Table S1. Association between the month of blood collection (sunny vs. non-sunny) and SLEDAI-2K score.

Disease activity (SLEDAI-2K score)	Month of blood collection						p
	Non-sunny		Sunny		Total		
	n	%	n	%	n	%	
Low (≤6)	17	80.9	19	63.3	36	70.6	0.174
High (>6)	4	19.1	11	36.7	15	29.4	
Total	21	100.0	30	100.0	51	100.0	

Similarly, the prevalence of anti-dsDNA antibodies was slightly higher in sunny months than in non-sunny months (16.7% vs 14.3%), but this difference was not statistically significant ($\chi^2 = 0.050$; $p > 0.05$). Logistic regression did not show a significant effect of season on the risk of anti-dsDNA seropositivity (OR = 1.20; 95% CI: 0.243–5.906; $p = 0.819$). (Supplementary Table S2).

Supplementary Table S2. Association between the month of blood collection (sunny vs. non-sunny) and anti-dsDNA seropositivity.

dsDNA status	Month of blood collection						p
	Non-sunny		Sunny		Total		
	n	%	n	%	n	%	
Negative	18	85.7	25	83.3	43	84.3	0.818
Positive	3	14.3	5	16.7	8	15.7	
Total	21	100.0	30	100.0	51	100.0	

2. *The effect of vitamin D supplementation on its concentration, disease activity and anti-dsDNA antibodies*

Vitamin D supplementation had a significant impact on achieving normal serum 25(OH)D concentrations. Among patients supplementing vitamin D, sufficient concentrations of this vitamin were found in 62.9% of patients, compared to only 18.8% in the group without supplementation. This difference was statistically significant ($\chi^2 = 8.550$; $p < 0.05$). Univariate regression analysis showed that vitamin D supplementation increased the odds of achieving normal 25(OH)D levels more than sevenfold (OR = 7.33; 95% CI: 1.692–31.789; $p = 0.009$) (Supplementary Table 3).

Supplementary Table S3. Association between vitamin D supplementation and its concentration in patients with SLE.

Vitamin D concentration	Vitamin D supplementation						p
	No		Yes		Total		
	n	%	n	%	n	%	
<30 ng/mL	13	81.2	13	37.1	26	84.3	0.004
≥30 ng/mL	3	18.8	22	62.9	25	49.0	
Total	16	100.0	35	100.0	51	100.0	

However, no significant correlation was found between vitamin D supplementation and disease activity. High SLE activity was observed in 31.4% of patients supplementing vitamin D and in 25.0% of patients without supplementation ($\chi^2 = 0.210$; $p > 0.05$). Supplementation was associated with a statistically insignificant increase in the risk of high disease activity (OR = 1.38; 95% CI: 0.349–5.429; $p = 0.643$) (Supplementary Table S4).

Supplementary Table S4. Association between vitamin D supplementation and SLEDAI-2K score.

Disease activity (SLEDAI-2K score)	Vitamin D supplementation						p
	No		Yes		Total		
	n	%	n	%	n	%	
Low (≤6)	12	75.0	24	68.6	36	70.6	0.643
High (>6)	4	25.0	11	31.4	15	29.4	
Total	16	100.0	35	100.0	51	100.0	

Similarly, vitamin D supplementation had no significant effect on the presence of anti-dsDNA antibodies. These antibodies were present in 17.1% of patients receiving supplementation and in 12.5% of patients without supplementation ($\chi^2 = 0.180$; $p > 0.05$) and logistic regression showed no significant association (OR = 1.45; 95% CI: 0.248–8.472; $p = 0.675$) (Supplementary Table S5).

Supplementary Table S5. Association between vitamin D supplementation and anti-dsDNA antibody positivity in patients with SLE.

dsDNA status	Vitamin D supplementation						p
	No		Yes		Total		
	n	%	n	%	n	%	
Negative	14	87.5	29	82.9	43	84.3	0.675
Positive	2	12.5	6	17.1	8	15.7	
Total	16	100.0	35	100.0	51	100.0	

3. *Lifestyle factors and their association with disease activity and anti-dsDNA antibodies*

Additional analyses were performed to evaluate the association of potential confounding variables (age, sex, smoking, diet, season of blood sampling, and vitamin D supplementation) with disease activity and anti-dsDNA status. None of these variables showed statistically significant associations with SLEDAI category or with anti-dsDNA

positivity (all $p > 0.05$), and post hoc power for most comparisons ranged between 2% and 30%. These results indicate that the distribution of key covariates was similar across outcome groups, reducing the likelihood of major confounding effects.

No statistically significant correlation was found between special diet, including lactose-free, gluten-free, low-fat, diabetic and Mediterranean diet, and disease activity or the presence of anti-dsDNA antibodies. High SLE activity was observed in 28.6% of patients following the diet and in 29.6% of those not following it ($\chi^2 = 0.155$; $p = 0.694$) (Supplementary Table S6). Similarly, the prevalence of anti-dsDNA antibodies was comparable in both groups (14.3% vs. 15.9%; $\chi^2 = 0.202$; $p = 0.653$) (Supplementary Table S7). Univariate regression analysis showed no significant effect of diet on disease activity or anti-dsDNA seropositivity.

Supplementary Table S6. Association between diet and SLEDAI-2K score in patients with SLE.

Disease activity (SLEDAI-2K score)	Diet						p
	No		Yes		Total		
	n	%	n	%	n	%	
Low (≤6)	31	70.4	5	71.4	36	70.6	0.694
High (>6)	13	29.6	2	28.6	15	29.4	
Total	44	100.0	7	100.0	51	100.0	

Supplementary Table S7. Association between diet and anti-dsDNA antibody positivity in patients with SLE.

dsDNA status	Diet						p
	No		Yes		Total		
	n	%	n	%	n	%	
Negative	37	84.1	6	85.7	43	84.3	0.653
Positive	7	15.9	1	14.3	8	15.7	
Total	44	100.0	7	100.0	51	100.0	

Smoking also had no significant effect on the parameters studied. High disease activity was more common in smokers than in non-smokers (40.0% vs 25.0%), but this difference was not statistically significant ($\chi^2 = 1.148$; $p = 0.284$) (Supplementary Table S8). The presence of anti-dsDNA antibodies was less common in smokers (6.7%) than in non-smokers (19.4%), but this difference was also not statistically significant ($\chi^2 = 0.520$; $p = 0.471$) (Supplementary Table S9). Logistic regression did not confirm a significant effect of smoking on disease activity or anti-dsDNA seropositivity.

Supplementary Table S8. Association between smoking and SLEDAI-2K score.

Activity (SLEDAI-2K score)	Smoking						p
	No		Yes		Total		
	n	%	n	%	n	%	
Low (≤6)	27	75.0	9	60.0	36	70.6	0.284
High (>6)	9	25.0	6	40.0	15	29.4	
Total	36	100.0	15	100.0	51	100.0	

Supplementary Table S9. Association between smoking and anti-dsDNA antibody positivity in patients with SLE.

dsDNA status	Smoking						p
	No		Yes		Total		
	n	%	n	%	n	%	
Negative	29	80.6	14	93.3	43	84.3	0.471
Positive	7	19.4	1	6.7	8	15.7	
Total	36	100.0	15	100.0	51	100.0	

4. *Analysis of the Relationship between Disease Phenotype, Treatment and Vitamin D level*

A descriptive analysis was performed to explore the relationship between serum vitamin D levels, predominant disease domain, and current pharmacotherapy (Supplementary Tables S10 and S11).

Mean 25(OH)D concentrations across individual SLE phenotypes showed moderate variability—from the lowest values among patients with concurrent cutaneous and immunological involvement (approximately 18–25 ng/mL) to the highest levels in those with joint manifestations (around 42 ng/mL) or limited solely to the immunological domain (about 36 ng/mL). However, due to the very small number of patients in each subgroup (1–8 individuals), it was not possible to perform a valid statistical comparison between phenotypes.

Analysis by therapeutic regimen also revealed a wide range of mean values (18–60 ng/mL). The lowest mean 25(OH)D concentrations were observed in patients receiving a combination of antimalarial drugs and mycophenolate mofetil, whereas the highest level was recorded in a single patient treated with methotrexate (59.5 ng/mL). Among patients using glucocorticoids, the mean vitamin D concentration remained within 25–37 ng/mL. These values suggest that pharmacotherapy may exert a varied but quantitatively indeterminate influence on vitamin D status.

Overall, no clear trend indicating lower vitamin D levels in any specific phenotype or treatment regimen was observed, confirming that the clinical and therapeutic heterogeneity of the study population limited the ability to detect statistically significant associations.

Supplementary Table S10. Serum vitamin D levels across clinical phenotypes of SLE.

Clinical phenotype (domain)	n	Mean 25(OH)D (ng/mL)
Hematological, immunological	2	36.6
Hematological, cutaneous	4	25.15
Hematological, cutaneous, immunological	1	19.0
Hematological, cutaneous, articular, immunological	1	23.1
Hematological, articular, immunological	1	40.2
Hematological, articular, immunological, cutaneous	1	36.8
Immunological	8	36.43
Immunological, cutaneous	2	20.95
Immunological, mucosal	1	32.3
Renal, immunological	1	32.9
Cutaneous	4	30.85
Cutaneous, immunological	2	18.4
Cutaneous, immunological, hematological	1	24.6

Cutaneous, renal, immunological	2	24.0
Cutaneous, articular, immunological	1	36.2
Articular	2	42.1
Articular, immunological	2	22.41
Mucosal	1	32.0
Mucosal, cutaneous	1	24.9
Ocular, muscular	1	23.0
Cutaneous, articular	4	24.48

Supplementary Table S11. Serum vitamin D levels according to treatment regimens. Abbreviations: AM – antimalarial drugs, GKS – glucocorticoids, MM - mycophenolate mofetil, AZA – azathioprine, MTX – methotrexate

Treatment regimen	n	Mean 25(OH)D (ng/mL)
AM	14	28.005
AM + GKS	13	33.69
AM + MM	2	18.4
AZA + AM	2	25.7
AZA + GKS	2	27.15
GKS	3	25.1
GKS + AM + MM	4	26.6
GKS + AM + MTX	2	28.05
GKS + AZA + AM	2	28.2
GKS + MM	1	36.2
GKS + MTX	2	36.55
MTX	1	59.5

5. *Association of CRP with vitamin D status and disease activity*

Serum CRP concentrations were additionally analysed as a marker of systemic inflammation. No significant correlation was observed between CRP and serum 25(OH)D concentration (Spearman's $\varrho = -0.063$, $p = 0.662$; Supplementary Table S12) or between CRP and disease activity assessed by SLEDAI-2K ($\varrho = 0.009$, $p = 0.952$; Supplementary Table S12). Similarly, anti-dsDNA seropositivity was not significantly associated with elevated CRP levels (>5 mg/L) (p = 0.785; Supplementary Table S13).

Supplementary Table S12. Spearman correlation analysis of serum CRP concentration with serum 25(OH)D levels and SLEDAI-2K score in patients with SLE.

Association	Spearman's rank correlation coefficient ϱ	p
CRP & Vitamin D	-0,063	0,6621
CRP & SLEDAI-2K	0,009	0,9524

Supplementary Table S13. Association of elevated CRP levels (>5 mg/L) with anti-dsDNA seropositivity in patients with SLE.

dsDNA status	CRP≤5 mg/l	CRP>5 mg/l	χ^2	p
Negative	29 (85.3%)	14 (82.4%)	0.074	0.785
Positive	5 (14.7%)	3 (17.6%)		
Total	34 (100.0%)	17 (100.0%)		
